# Self-Perceived Preparedness Needs Among Caregivers of Veterans With and Without Dementia: An Exploratory Study Using Open-Ended Survey Data

**DOI:** 10.2196/83493

**Published:** 2026-01-22

**Authors:** Roshni Singh, Sandra Garcia-Davis, Richard Munoz, Saanvi Lamba, Diana Ruiz, Pranjal Tyagi, Erin Bouldin, Linda Nichols, Marianne Desir, Luci Leykum

**Affiliations:** 1 Miller School of Medicine University of Miami Miami, FL United States; 2 South Florida Veterans Affairs Foundation for Research and Education (SFVAFRE) Miami, FL United States; 3 Elizabeth Dole Center of Excellence for Veteran and Caregiver Research US Department of Veterans Affairs San Antonio, TX United States; 4 Department of Public Health Dr Kiran C Patel College of Osteopathic Medicine Nova Southeastern University Fort Lauderdale, FL United States; 5 Flint Hill School Oakton, VA United States; 6 Miami Veterans Affairs Healthcare System Miami, FL United States; 7 Informatics, Decision Enhancement, and Analytic Sciences Center (IDEAS) VA Salt Lake City Health Care System Salt Lake City, UT United States; 8 Department of Internal Medicine Spencer Fox Eccles School of Medicine University of Utah Salt Lake City, UT United States; 9 Department of Preventive Medicine University of Tennessee Health Science Center Memphis, TN United States; 10 VA Caregiver Center Lt. Col. Luke Weathers, Jr. Veterans Affairs Medical Center Memphis, TN United States; 11 Miami VA Healthcare System Geriatric Research Education and Clinical Center (GRECC) Miami, FL United States; 12 Department of Family Medicine and Community Health Miller School of Medicine University of Miami Miami, FL United States; 13 Herbert Wertheim College of Medicine Florida International University Miami, FL United States; 14 South Texas Veterans Health Care System San Antonio, TX United States; 15 Dell Medical School University of Texas at Austin Austin, TX United States

**Keywords:** caregiver preparedness, dementia, caregiver, veterans, survey, caregiver burden, unmet needs, care coordination

## Abstract

**Background:**

Caregivers’ self-perceived preparedness for caregiving influences care recipients’ and caregivers’ emotional health, and care recipients’ aging in place. Dementia’s unique, long, and progressive nature compared to other age-related illnesses, along with associated behavioral symptoms and personality changes, may cause caregivers’ preparedness to vary significantly from that of those caring for patients with other chronic conditions.

**Objective:**

This study aimed to describe and compare specific domains and tasks in which family caregivers of veterans with and without dementia reported wanting to be better prepared.

**Methods:**

Using the Veterans Affairs’ HERO CARE (Home Excellence Resource Outcome Center to Advance, Redefine, and Evaluate Non-Institutional Care) Survey data, we analyzed caregivers’ responses to one open-ended question: “Out of all the tasks that you help the veteran with, is there anything specific you would like to be better prepared for?” Response themes were deductively coded into 9 domains, and differences in reported domains between caregivers of care recipients with and without dementia were compared.

**Results:**

A total of 732 caregivers were included: 301 (41.1%) caregivers of veterans with dementia and 431 (58.9%) without. Caregivers of veterans with and without dementia, respectively, were similar except in age, being spousal caregivers, working at least part-time, hours of care provision per week, and proportion with a high burden. Veterans with dementia, versus without, were older and had higher frailty and risk scores. Preparedness concerns among caregivers (N=732) included care coordination (n=164, 22.4%), emotional and social support (n=145, 19.8%), advance planning (n=116, 15.8%), nursing and health monitoring (n=94, 12.8%), personal care (n=65, 8.9%), mobility (n=79, 10.8%), household (n=58, 7.9%), caregiver self-care (n=36, 4.9%), and emergent situations (n=28, 3.8%). The commonest tasks caregivers expressed needs for included managing emotional and behavioral symptoms (n=74, 10.1%), recognizing and responding to significant changes in the veterans’ condition (n=66, 9.0%), seeking medical information relevant to the veterans’ needs (n=54, 7.4%), handling financial and legal matters (n=52, 7.1%), and advocating for services (n=49, 6.7%). Similar proportions of caregivers of veterans with and without dementia reported preparedness needs in all domains and tasks.

**Conclusions:**

The preparedness needs of caregivers of veterans with and without dementia were mostly similar in most domains and tasks. The commonest preparedness gaps were in the domains of care coordination, emotional and social support, and advance planning. The findings can inform interventions to prepare all caregivers to support aging in place.

## Introduction

Persons with complex chronic illnesses, including dementia, experience a wide range of functionally limiting needs, many of which are met by family caregivers. Caregivers are defined here as friends and family members who are involved in the care of patients in community-based settings. Family caregivers support their care recipients in many ways and are critical to their safe aging at home. These caregivers often manage their care recipients’ needs and tasks, related to self-care, monitoring medical conditions, and providing emotional and social support [1].

Caregiver preparedness is defined as “how ready caregivers believe they are for the tasks and stresses of caregiving” [2-4]. It includes caregivers’ perceived ability to meet the physical and emotional needs, care coordination, and emergency needs of their care recipients [5]. Prior studies have shown that better caregiver preparedness is associated with improved outcomes for the dyad: caregivers who feel more prepared have lower burden, strain, and depression [6-8], and have better self-care [9]. Care recipients, in return, have decreased risks of hospital readmission [10], higher quality of life [11], better recovery [12], and lower caregivers’ desire to institutionalize their care recipient [13]. Therefore, caregiver preparedness is a critical area of concern in health care systems, since prepared caregivers can positively impact the health of both their care recipients and caregivers. While dyadic outcomes can improve with better preparedness, many caregivers do not receive training for their role [14]. The “Home Alone” study found that more than 60% of caregivers learn how to manage medical and nursing tasks on their own, with 47% never receiving training from any source [15]. In the report “Caregiving in the U.S. 2015,” more than 40% of the caregivers reported performing medical or nursing tasks without any preparation, and only 14% reported having received some preparation [16].

While the onset and progression of the caregiving roles vary widely depending on the type and cause of disability, tasks and responsibilities generally are grouped into several broad themes such as household, nursing and medical tasks, advocacy, and care coordination. Several studies have categorized family caregiving tasks in general [1,17-19] and of caregivers caring for care recipients with dementia [20-22]. Caregivers of people with dementia, as a subgroup, garner special attention for several reasons: first, one-third of family and unpaid caregivers for noninstitutionalized older adults care for care recipients with dementia; second, dementia often demands more intensive support over extended periods of time compared to caring for care recipients without dementia [22,23] and involves assisting not only with physical health needs, but also managing behavioral symptoms and change in personality [24]; third, studies show disproportionately higher levels of burden among caregivers caring for care recipients with dementia compared to other conditions [25-28]; and forth, due to the progressive, evolving nature of dementia [25], caregivers may experience a significant lack of preparedness leading to care recipients’ poor health outcomes and higher likelihood of early institutionalization and mortality [29].

Therefore, caregiving for care recipients with dementia is often considered intrinsically different from other chronic diseases, and caregivers caring for veterans with dementia may have different preparedness needs compared to other caregivers. Yet, to our knowledge, no studies have examined the differences in preparedness gaps between family caregivers of veterans with and without dementia.

This study aimed to describe specific domains and tasks in which family caregivers want to be better prepared and to compare preparedness gaps of caregivers caring for veterans with and without dementia. We hypothesized that caregivers of veterans with dementia would express a greater need for preparedness in domains related to managing behavioral symptoms, advance planning, and caregiver self-care.

## Methods

### Ethical Considerations

This project was classified as a nonresearch project by the Miami Veterans Affairs Healthcare System Human Studies Subcommittee because the findings were intended to inform VA (Veteran Affairs) operations and were therefore exempt from institutional review board review in compliance with VA Handbook 1058.05i. A waiver of documentation of authorization and informed consent was obtained. No compensation was provided to veterans for participation. To ensure privacy, efforts were taken to protect the identity of participants and ensure that data were kept confidential. Identifiable information will only be maintained on a VA server; documentation of the procedure used to code the data will remain within the VA. All identifiers collected as part of this project will be destroyed as per the records control schedule (RCS 10-1) of the Veterans Health Administration. Electronic files will be stored in folders with restricted access on a protected computer shared drive behind the VA firewall in a secure server. Data will not be transmitted as an attachment to unprotected email messages. The data will be accessible only to personnel involved in the study. All staff were trained to avoid breaches of confidentiality.

### Study Design and Participants

We used cross-sectional data from the Veterans Affairs’ HERO CARE (Home Excellence Resource Outcome Center to Advance, Redefine, and Evaluate Non-Institutional Care) Caregiver survey, administered from July 2021 to January 2022. The caregiver survey was included in a veteran survey packet mailed to 20,000 veterans across 4 VA sites (Miami, FL; Palo Alto, CA; Salt Lake City, UT; San Antonio, TX), and 1 Veteran Integrated Service Network (VISN 8). We oversampled veterans with a higher predicted 2-year long-term institutional care risk [30]. Detailed survey methods have been previously delineated [31].

Here, we specifically looked at responses to the open-ended question: “Is there anything specific you would like to be better prepared for?” Participants included caregivers (1) who had responded to the open-ended question and (2) who cared for a veteran for whom the VA had a Geriatrics and Extended Care Data Analysis Center Core Files (GCF) [32] electronic medical record that allowed classifying their dementia status. Using responses to this open-ended question, we identified tasks for which family caregivers felt insufficiently prepared for, grouping them by the veterans’ dementia status.

### Data Collection

We linked caregiver surveys to their Veterans’ GCF [32,33]. The GCF provides information on the veterans’ demographics, risk scores, comorbidities, and health care utilization. Veterans’ dementia status was pulled from VA electronic health record data using the Hierarchical Condition Category (HCC) codes HCC-51 (dementia with complications) and HCC-52 (dementia without complications). Veteran data regarding age, JEN Frailty Index (JFI) [34], and HCC count [35] were also similarly extracted.

Caregiver’s sociodemographic data were collected from the HERO CARE Caregiver survey responses and included many domains: age, race and ethnicity, sex, preferred language, marital status, education level, health literacy [36], employment, and financial insecurity [37]. Low health literacy was defined by responses of “somewhat,” “a little,” or “not at all” to confidence in filling medical forms [36]. Financial security was a 1-item assessment asking, “Without giving exact dollars, how would you describe your household’s financial situation right now?” [37].

Caregiving characteristics included whether the caregiver was the veteran’s spouse, the primary, secondary, or shared caregiver, the veteran’s power of attorney, along with caregiving duration, and hours of care per week. Caregiver burden was measured using the measured using the 4-item Zarit Burden Interview screen, with scores summed across all four items for a total score ranging. “High” caregiver burden was attributed to a score of at least 8, per the scoring instructions [38,39].

### Analysis

We report descriptive statistics for caregiver sociodemographic characteristics and health measures overall, by veterans’ dementia status. Frequencies and percentages are reported for categorical variables using chi-square tests and means and SDs for continuous variables using independent *t* tests.

Qualitative content analysis from the open-ended survey responses consisted of deductively coding and organizing responses from all caregivers into broad themes representing domains such as mobility or personal care, and subthemes representing tasks within a domain such as assisting with dressing and bathing or with toileting, etc. The coding was first done by two authors (RS and SL) independently, and discrepancies were resolved by a third author (DR), with further discussion and modifications with the rest of the team until agreement was reached. If a caregiver reported preparedness needs in more than one domain, they were documented and counted in both domains [40]. If they reported preparedness needs for more than one task in a specific domain, we documented both tasks, but the caregiver was only counted once in that domain, to try to accurately reflect the number of caregivers who had needs in a specific domain.

After classifying responses under each domain (theme) and subdomain (tasks), we summed them and grouped them by veterans’ dementia status. Frequency and percentages of responses by dementia status of veterans were reported by domain and tasks [40].

### Caregiver Preparedness Framework

After a preliminary review of the emerging themes, we decided to sort them into predefined domains from the University of California Davis (UC Davis) Health’s Family Caregiver Domains of Preparedness, since that framework captured most of the themes and subthemes we identified [3]. It consists of 9 broad domains of activities that characterize family caregiving, and each domain comprises several tasks. After a preliminary review of the emerging themes from the survey responses, we modified this existing framework in the following ways. We combined health monitoring with nursing and management of behavioral symptoms with emotional and social support. We also created two new categories: (1) emergent situations, to recognize and respond to significant changes in the veterans’ medical condition and medical situations related to weather, and (2) advance planning, combining shared decision-making with concerns regarding end-of-life. The domains in our study included household tasks, personal care, mobility, emotional and social support, care coordination, nursing and health monitoring, advance planning, emergent situations, and caregiver self-care. Domains and subdomains or tasks are shown in Textbox 1.

**Domains and tasks in each domain for which caregivers wanted to be better prepared.**

**Care coordination**

- Navigate and communicate within the health care system (doctors, nurses, social workers, pharmacists, and other health care and long-term services and supports).
- Seek medical information relevant to the care recipient’s needs
- Participate in treatment decisions
- Advocate for services
- Locate and arrange resources for cash, food, and transportation

**Personal care**

- Assist with bathing, dressing, feeding, grooming, and personal hygiene
- Assist with toileting (eg, getting to and from the toilet, managing incontinence episodes, and maintaining continence)
- Arrange and manage in-home help

**Mobility**

- Assist the care recipient to safely transfer in or out of bed, chair, wheelchair, toilet, and tub or shower
- Home modifications
- Manage assistive devices such as walkers, canes, or wheelchairs
- Assist with appropriate mobility and strengthening exercises

**Emotional and social support**

- Provide support in managing stressful situations
- Manage emotional and behavioral symptoms

**Advance planning**

- Participate advance planning (long-term care placement “future”)
- End of lifecare hospice, palliative
- Handle financial and legal matters

**Nursing and health monitoring tasks**

- Support treatment adherence
- Administer medications including oral, topical, and injectable
- Provide wound care
- Manage healthy sleep hygiene
- Manage physical symptoms (eg, nausea, pain, and constipation)
- Use medical devices to monitor patients’ condition (eg, blood pressure cuff and pulse oximeter)
- Manage hearing or vision deficits
- Access training resources (nursing training, certified nursing assistant, cardiopulmonary resuscitation, health monitoring, fall prevention, and equipment use)

**Household tasks**

- Assist with paying bills and managing finances
- Manage laundry, prepare meals, perform shopping, and run errands
- Perform or coordinate home maintenance activities, including odd jobs

**Caregiver self-care**

- Engage in activities that support caregivers’ own mental, emotional, and physical well-being
- Ask for and accept assistance (eg, respite care)

**Emergent situations**

- Medical emergencies
- Weather-related emergencies (hurricanes and power outages)

## Results

### Final Sample

**Figure 1:**
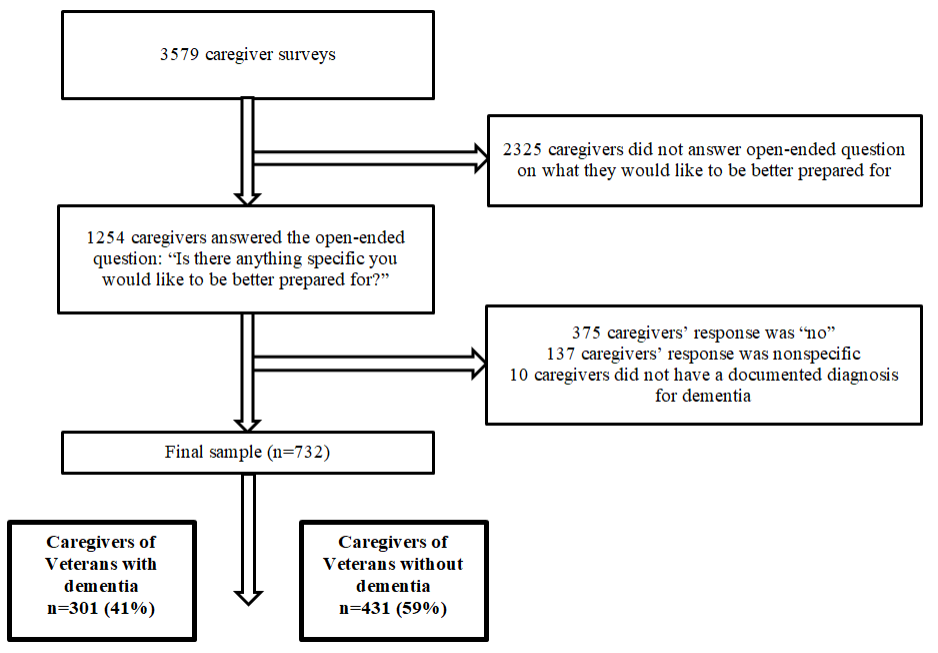
CONSORT (Consolidated Standards of Reporting Trials) flow diagram.

Of the 3579 caregivers who responded to the HERO CARE Caregiver survey, 1254 caregivers answered the open-ended question: “Is there anything specific you would like to be better prepared for?” We excluded caregivers whose response was a “no” (n=375) or was nonspecific (n=137). We also excluded caregivers whose veterans did not have positive or negative documentation for a dementia diagnosis (n=10). Our final sample consisted of 732 caregivers: 301 (41.9%) caregivers of veterans with dementia and 431 (58.9%) caregivers of veterans without dementia (Figure 1).

### Veteran and Caregiver Characteristics

The sociodemographic characteristics of the caregivers and veterans overall, and by veterans’ dementia status, are given in Table 1. Caregivers of veterans with (n=301) and without (n=431) dementia, respectively, were similar except in age (71 vs 66 years; *P*<.001), being spousal caregivers (203/301, 67.4% vs 242/431, 56.1%; *P*=.004), working at least part-time (47/301, 15.6% vs 98/431, 22.7%; *P*=.003), hours of care provision per week (mean 94.2, SD 65.9 vs mean 75.0, SD 63.1; *P*<.002), and proportion with a high burden (162/301, 53.8% vs 170/431, 39.9%; *P*<.001) based on a Zarit Burden Interview score of ≥8 [39]. Veterans with dementia versus without, respectively, were older (mean 82.4, SD 7.8 vs mean 78.4, SD 10.7 years; *P*<.001), had higher US Centers for Medicare & Medicaid Services’ HCC risk scores (mean 2.6, SD 1.6 vs mean 2.2, SD 1.5; *P*<.001), and higher JFI (mean 6.4, SD 2.2 vs mean 5.3, SD 2.4; *P*<.001).

**Table 1:** Sociodemographic characteristics overall and by veterans’ dementia status^a^.

				Overall (N=732)		Veterans with dementia (n=301)		Veterans without dementia (n=431)		*P* value	
**Caregiver characteristics**											
	Age in years, mean (SD)		68.1 (12.5)		71.0 (11.0)		66.0 (13.0)		<.001		
	**Respondent, n (%)**										.34
		Caregiver (self)	556 (76.0)		230 (76.4)		326 (75.6)				
		Caregiver with help	60 (8.2)		21 (7.0)		39 (9.0)				
	**Race and ethnicity, n (%)**										.25
		Non-Hispanic White	508 (69.4)		220 (73.1)		288 (68.8)				
		Non-Hispanic Black	59 (8.1)		19 (6.3)		40 (9.3)				
		Hispanic	107 (14.6)		41 (13.6)		66 (15.3)				
		Other	41 (5.6)		14 (4.7)		27 (6.3)				
	**Sex, n (%)**										.99
		Female	631 (86.2)		260 (86.4)		371 (86.1)				
		Male	91 (12.4)		37 (12.3)		54 (12.5)				
	**English as preferred language, n (%)**										.04
		Yes	696 (95.1)		279 (92.7)		417 (96.8)				
		Other	33 (4.5)		20 (6.6)		13 (3.0)				
	**Marital status, n (%)**										<.001
		Married, in a civil union or domestic partnership, or living with a partner	570 (77.9)		259 (86.0)		311 (72.2)				
		Not married	144 (19.7)		39 (13.0)		105 (24.4)				
	**Education, n (%)**										.54
		High school graduate, general education development certification or less	181 (24.7)		72 (23.9)		109 (25.3)				
		Associate’s degree or small college credit	290 (39.6)		117 (38.9)		173 (40.1)				
		Bachelor's degree or graduate school	220 (30.1)		98 (32.6)		122 (28.3)				
	**Low health literacy, n (%)**										.74
		Yes	624 (85.2)		259 (86.0)		365 (84.7)				
		No	103 (14.1)		41 (13.6)		62 (14.4)				
	**Caregiver employment, n (%)**										.003
		Working at least part-time	145 (19.8)		47 (15.6)		98 (22.7)				
		Not working	67 (9.2)		19 (6.3)		48 (11.1)				
		Homemaker	84 (11.5)		30 (10.0)		54 (12.5)				
		Retired	299 (40.8)		143 (47.5)		156 (36.2)				
		Other	47 (6.4)		21 (7.0)		26 (6.0)				
	**Caregiver employment changed, n (%)**										.31
		No	334 (45.6)		138 (45.8)		196 (45.5)				
		Yes	266 (36.3)		99 (32.9)		167 (38.7)				
	**Income, n (%)**										.06
		After paying the bills, you still have enough money for special things that you want	254 (34.7)		109 (36.2)		145 (33.6)				
		You have enough money to pay the bills, but little spare money to buy extra or special things	232 (31.7)		99 (32.9)		133 (30.9)				
		You have money to pay the bills, but only because you have to cut back on things	112 (15.3)		40 (13.3)		72 (16.7)				
		You are having difficulty paying the bills, no matter what you do	46 (6.3)		11 (3.7)		35 (8.1)				
	**Caregiver-veteran relationship, n (%)**										.004
		Spousal	445 (60.8)		203 (67.4)		242 (56.1)				
		Other	259 (35.4)		89 (29.6)		170 (39.4)				
	**Caregiver as primary caregiver, n (%)**										.45
		Yes	620 (84.7)		254 (84.4)		366 (84.9)				
		Someone else is the primary	25 (3.4)		13 (4.3)		12 (2.8)				
		Share caregiving equally	66 (9.0)		28 (9.3)		38 (8.8)				
	**Hours of care provision per week, mean (SD)**										.002
		Caregiver hours of care/week	83.0 (64.9)		94.2 (65.9)		75.0 (63.1)				
	**Caregiving length of time, n (%)**										.13
		Less than 6 months	18 (2.5)		4 (1.3)		14 (3.2)				
		6-11 months	35 (4.8)		11 (3.7)		24 (5.6)				
		1-2 years	108 (21.3)		41 (13.6)		67 (15.5)				
		3-5 years	156 (21.3)		75 (24.9)		81 (18.8)				
		More than 5 years	391 (53.4)		158 (52.5)		233 (54.1)				
	**Zarit Caregiver Burden^b^**										.002
		Zarit Caregiver Burden Score, mean (SD)	7.0 (4.1)		7.8 (4.2)		6.4 (3.9)		<.001		
		Low to Moderate Caregiver Burden (0-7), n (%)	398 (54.4)		139 (46.2)		259 (60.1)		—^c^		
		High Caregiver Burden (8-16), n (%)	334 (45.6)		162 (53.8)		170 (39.9)		—		
**Veteran characteristics, mean (SD)**											
	Age in years		80.1(9.8)		82.4 (7.8)		78.4 (10.7)		<.001		
	CMS-HCC risk score^d^		2.4 (1.6)		2.6 (1.6)		2.2 (1.5)		<.001		
	JFI^e^		5.8(2.4)		6.4 (2.2)		5.3 (2.4)		<.001		
	Nosos		2.3 (2.3)		2.5 (2.3)		2.2 (2.3)		.05		

^a^Percents do not include missing. Missingness is as follows: respondent type (n=116), race and ethnicity (n=17), sex (n=10), preferred language (n=3), marital status (n=18), education (n=41), heath literacy (n=5), caregiver employment (n=90), change in employment (n=132), income (n=88), caregiver-veteran relationship (n=28), primary caregiver status (n=21), caregiving length of time (n=24).

^b^Caregiver Burden Measured by the Zarit 4-item scale (scores of ≥8 indicate High Caregiver Burden) [39,40].

^c^Not applicable.

^d^CMS: U.S. Centers for Medicare & Medicaid Services; HCC: Hierarchical Condition Category. The CMS-HCC risk-adjusted score is the sum of the score or weight attributed to each of the demographic factors and HCCs used in the scoring model. The CMS-HCC model is normalized to 1.0, meaning veterans would be considered relatively healthy, and therefore less costly, with a risk score less than 1.0. HCCs are a risk-adjustment model used to estimate future health care costs for patients [35].

^e^The JFI or JEN Frailty Index (range 0-13) is calculated using 13 categories of ICD-9/10 (International Classification of Diseases) diagnostic codes representing geriatric syndromes, functional deficits, and multimorbidity clusters to predict the risk of nursing home admission [34].

### Qualitative Results for Caregiver Preparedness Needs

#### Overview of Emergent Themes and Domains

Below, we present the 9 domains derived from the themes that emerged from qualitative content analysis of open-ended responses and share a representative quote for each domain for a caregiver, each providing care to a veteran with and without dementia. Both sets of caregivers seemed to have similar preparedness needs across domains, and both often handled complex tasks spanning multiple domains and multiple tasks in one domain.

#### Care Coordination

The commonest concern expressed by caregivers was the need to learn how to arrange care and advocate for services and supports to make sure veterans got appropriate, timely help. They wanted to learn to navigate and communicate within the health care system and to participate in treatment decisions. They also wanted to understand how to coordinate services between different health care systems and to gather relevant information about available resources.

Working through getting services from the VA and coordination with PHI (private health insurance) and Medicare and long-term care insurance.

64-year-old non-Hispanic White, American Indian, or Alaska Native male caregiver of veteran with dementia

Sometimes paperwork, claims, or trying to get him at a higher percentage disability through system as there are no records of what my dad hauled/transported.

45-year-old Hispanic Asian or Asian American female caregiver of veteran without dementia

#### Emotional and Social Support

Family caregivers of both persons with and without dementia would benefit from more training to handle emotional outbursts, fluctuating mood, and agitation in the veterans, and provide support in managing stressful situations.

Dealing with his progressive fluctuating vascular dementia, dementia agitation and hostile combativeness on occasion.

54-year-old non-Hispanic White female caregiver of veteran with dementia

I need more training about how to deal with post-traumatic stress disorder, which my veteran husband has.

69-year-old Hispanic, some other race, or origin female caregiver of veteran without dementia

#### Advance Planning

Caregivers wanted to know how to keep the veterans at home for as long as possible, and how to arrange the needed long-term care for the veterans as their health, or that of the caregivers, declined. A common need was learning how to better handle financial and legal matters. Caregivers expressed worry about handling death and a desire to learn how to manage care at the end of life.

He and I don’t want him to go to a nursing home. How would I handle death alone at home?

78-year-old non-Hispanic White female caregiver of veteran with dementia

Long-term care when I can no longer lift him.

76-year-old non-Hispanic White female caregiver of veteran without dementia

#### Nursing and Health Monitoring Tasks

Caregivers wanted to learn how to properly administer oral, topical, and injectable medications, provide wound care, and support treatment adherence. They also wanted to know how to use medical devices to monitor the veterans’ condition (eg, blood pressure cuff and pulse oximeter), manage healthy sleep hygiene, and physical symptoms (eg, nausea, pain, and constipation). Some also expressed an interest in accessing various training courses, for example, nursing, nurse assistant, cardiopulmonary resuscitation, health monitoring, fall prevention, and equipment use.

Medical needs, medication management. Talking to the veteran about taking medicines/very poor compliance. Blood sugar dysregulated.

48-year-old non-Hispanic White male caregiver of veteran with dementia

Wound care, physical therapy, diapering, transfers. Not comfortable with the more medical issues...

58-year-old non-Hispanic White female caregiver of veteran without dementia

#### Household Tasks

Caregivers wanted to be better prepared to assist with household tasks like laundry, preparing meals, shopping, and performing or coordinating home maintenance activities, including odd jobs. They also wanted to be better prepared for assisting with paying bills and managing finances.

At my age (88) and having physical limitations, it is difficult to do all the cooking, driving, and laundry sometimes.

88-year-old non-Hispanic White female caregiver of veteran with dementia

I wish I were a superwoman so that I’ll be able to do all the household chores while taking care of my husband!

61-year-old non-Hispanic Asian or Asian American female caregiver of veteran without dementia

#### Personal Care

Caregivers wanted to learn how to better assist the veterans with personal care tasks like bathing, dressing, feeding, grooming, personal hygiene, toileting (including getting to and from the toilet), maintaining continence, and managing incontinence. They also wanted to know how to arrange and manage in-home help.

The emotional toll of caring for a bed-bound, urinary and bowel incontinent adult male. Even with a “hospital bed” I am not strong enough to turn him and move him to clean his bottom. The emotional stress of tube feeding. His inability to communicate effectively. Needing to make decisions for him.

68-year-old non-Hispanic White female caregiver of veteran with dementia

Toileting in bed, getting in and out of bed and chairs.

77-year-old non-Hispanic White female caregiver of veteran without dementia

#### Mobility

Caregivers wanted to be better prepared to assist the veterans with moving around inside or outside the home, and to safely transfer in or out of bed, wheelchair, toilet, and tub or shower, and prevent falls. They wanted to assist with appropriate mobility and strengthening exercises, and manage assistive devices such as walkers, canes, and wheelchairs, and access resources to make needed home modifications for safety. Caregivers wanted to learn how to maintain proper and safe body mechanics while assisting the veterans, to avoid injuring themselves.

Pulling and lifting, my wife injured her arm. Pulled tendons loose in her right arm.

76-year-old (unknown ethnicity) White male caregiver of veteran with dementia

How to make it easier for her to walk, sit, etc., without her being in so much pain and maybe falling. She can’t use her legs or hands good.

49-year-old non-Hispanic White female caregiver of veteran without dementia

#### Caregiver Self-Care

Caregiving tasks exact an extreme emotional toll on caregivers. Several caregivers reported feeling overwhelmed and needing emotional support and some time for themselves. They expressed interest in learning to engage in activities that support their own mental, emotional, and physical well-being. They wanted to learn how to ask for and accept assistance and access resources to get an occasional break, for example, respite care.

I can help at all things but wish I had a little time for me.

79-year-old non-Hispanic White female caregiver of veteran with dementia

I am needing assistance with an “adult (babysitter).” Someone to come in for weekend for about 4hrs a NEED day.

64-year-old non-Hispanic White female caregiver of veteran without dementia

#### Emergent Situations

Caregivers expressed concern about not being prepared for medical and weather-related emergencies and disasters (eg, hurricanes and power outages) and expressed a desire to proactively formulate plans for needed care and access to resources, equipment, and supplies during such times.

In the event of an emergency, to be able to get right help. If I am ill, to have proper care during my recovery.

85-year-old Hispanic White female caregiver of veteran with dementia

Would like to have a defibrillator machine at home.

49-year-old Hispanic White female caregiver of veteran without dementia

These results show that family caregivers often provide care for veterans with substantial impairments who need 24/7 nursing home-level care and require the caregivers to have skills in several domains and significant endurance. However, caregivers caring for veterans with and without dementia had similar preparedness needs.

### Quantitative Results for Caregiver Preparedness Needs

#### Overall Gaps in Caregiver Preparedness

**Figure 2:**
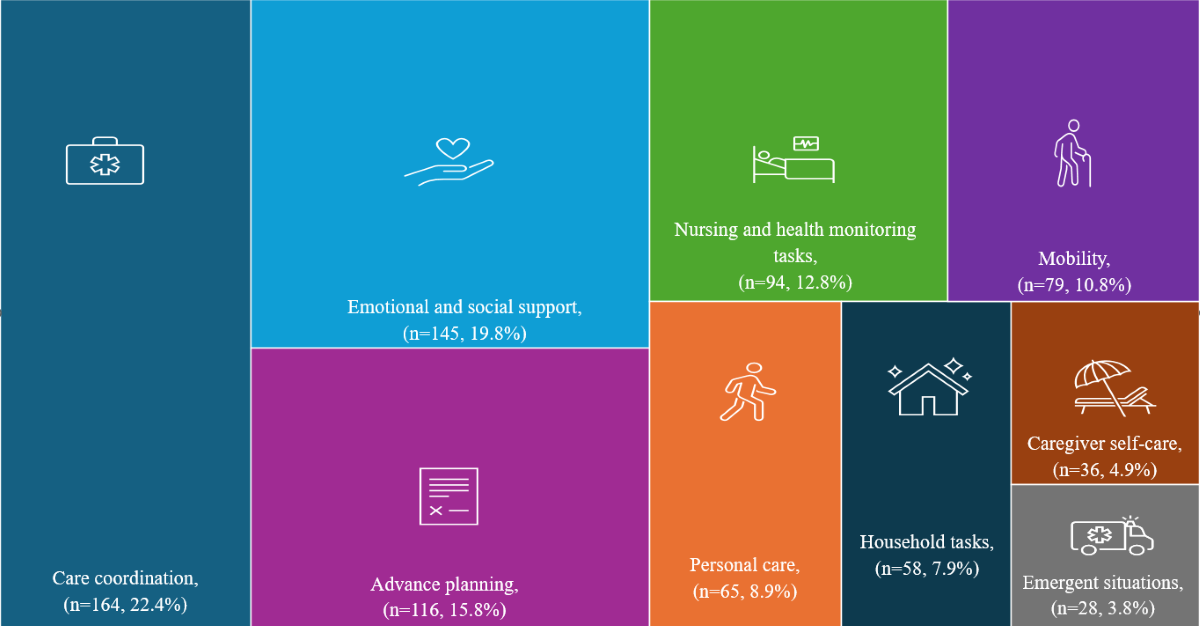
Preparedness needs by domain for all caregivers (N=732).

The number and proportion of all caregivers (n=732) who expressed preparedness needs by domain were care coordination 164 (22.4%), emotional and social support 145 (19.8%), advance planning 116 (15.8%), nursing and health monitoring tasks 94 (12.8%), mobility 79 (10.8%), personal care 65 (8.9%), household tasks 58 (7.9%), caregiver self-care 36 (4.9%), and emergent situations 28 (3.8%), and are shown in Figure 2.

#### Domains and Tasks for Which Caregivers Reported Preparedness Needs, by Veterans’ Dementia Status

**Figure 3:**
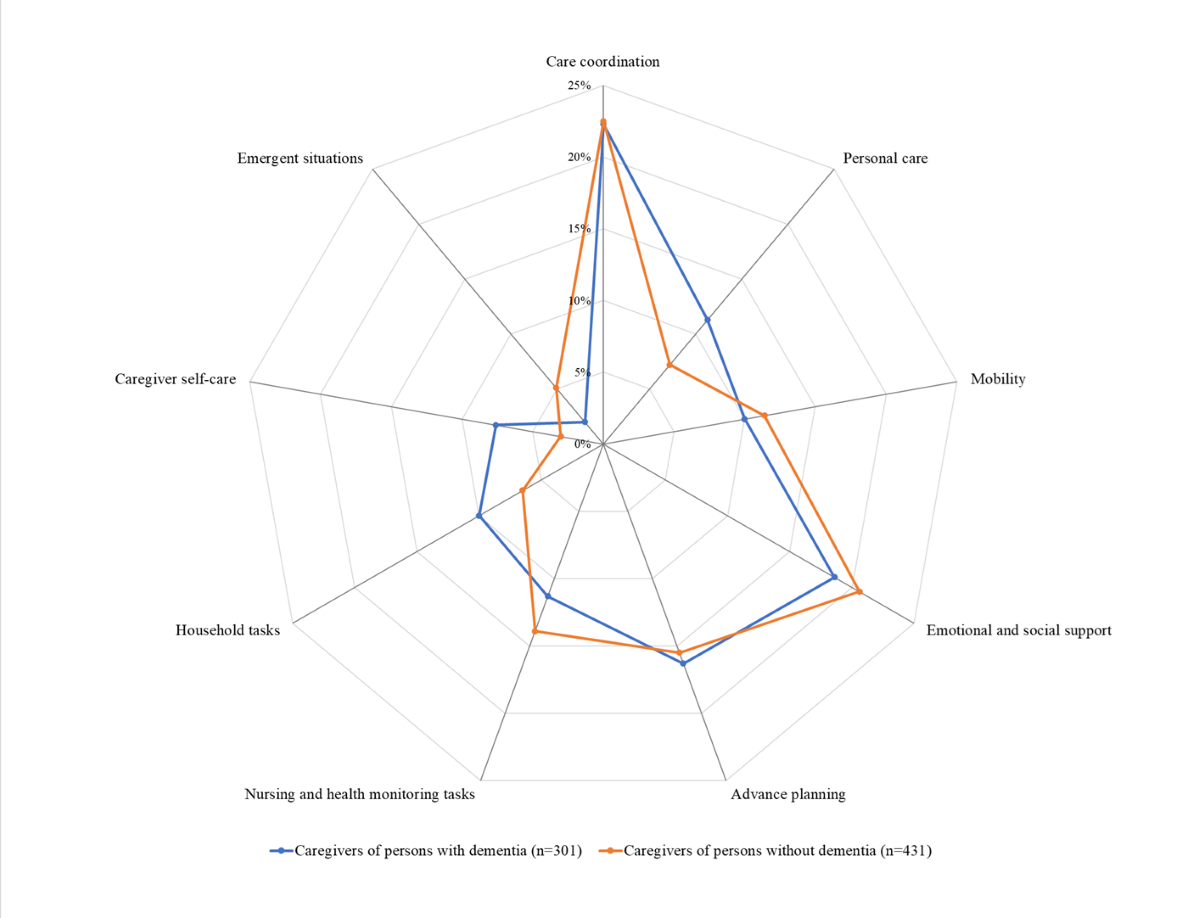
Preparedness needs of caregivers of veterans with and without dementia by domain.

**Figure 4:**
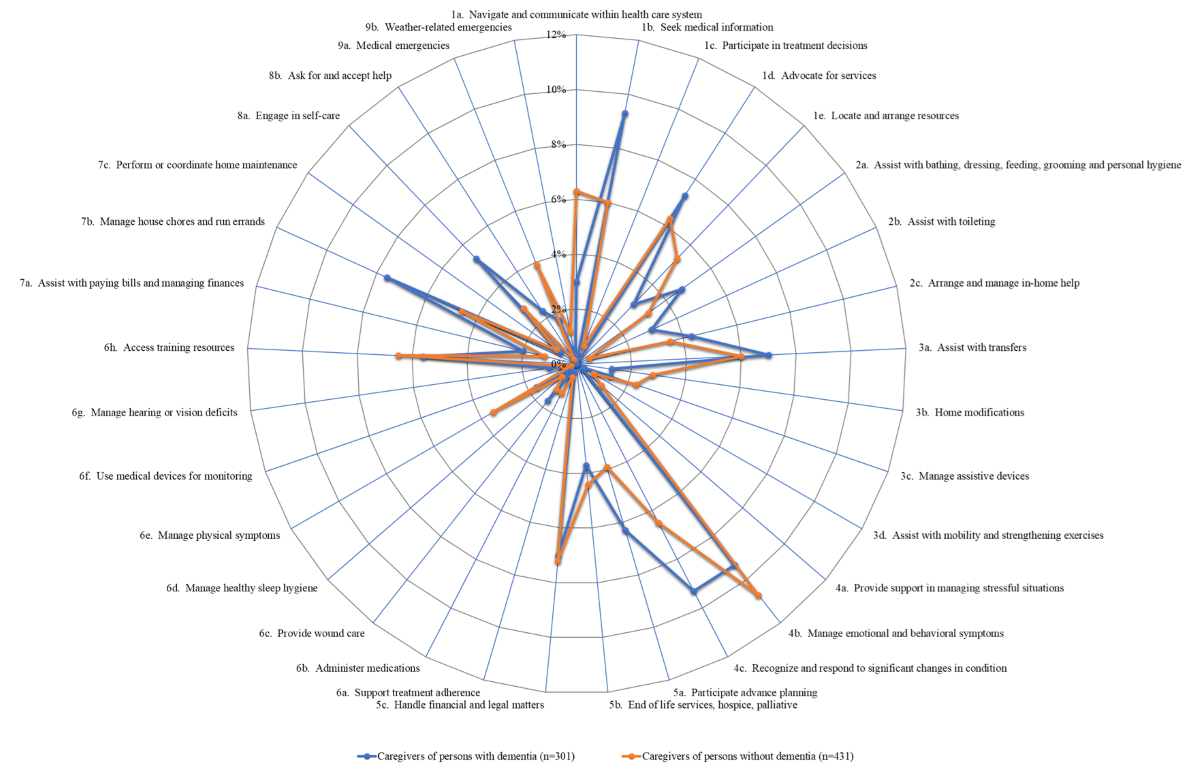
Preparedness needs for various tasks in each domain by dementia status. The tasks are organized by domains, with each number group representing a different domain. 1: coordination; 2: personal care; 3: mobility; 4: emotional and social support; 5: advance planning; 6: nursing and health monitoring tasks; 7: household tasks; 8: caregiver self-care; 9: emergent situations.

The number and proportion of caregivers reporting preparedness needs were tabulated next for each domain for caregivers of veterans with dementia (n=301) versus caregivers of veterans without dementia (n=431), respectively, and were as follows: care coordination: 67 (22.3%) versus 97 (22.5%); mobility: 30 (10%) versus 49 (11.4%); personal care: 34 (11.3%) versus 31 (7.2%); emotional and social support: 56 (18.6%) versus 89 (20.6%); advance planning: 49 (16.3%) versus 67 (15.5%); nursing and health monitoring: 34 (11.3%) versus 60 (13.9%); household tasks: 30 (10%) versus 28 (6.5%); caregiver self-care: 23 (7.6%) versus 13 (3%); and emergent situations: 6 (2%) versus 22 (5.1%). The comparisons are shown in Figure 3, with details in Multimedia Appendix 1. The tasks within each domain that caregivers wanted to be better prepared for, by dementia status, are shown in Figure 4. The commonest tasks caregivers overall (N=732) expressed needs for included managing emotional and behavioral symptoms 74 (10.1%), recognizing and responding to significant changes in the veterans” condition 66 (9.1%), seeking medical information relevant to the veterans’ needs 54 (7.4%), handling financial and legal matters 52 (7.1%), and advocating for services 49 (6.7%) (Table 2). Both Figures 3 and 4 show the overall similarity in the patterns of preparedness needs between caregivers of veterans with and without dementia.

**Table 2:** Commonest tasks caregivers want to be better prepared for overall and by veterans’ dementia status.

	Overall (N=732), n (%)	Caregivers of veterans with dementia (n=301), n (%)	Caregivers of veterans without dementia (n=431), n (%)
Manage emotional and behavioral symptoms	74 (10.1)	28 (9.3)	46 (10.7)
Recognize and respond to significant changes in the care recipient’s condition	66 (9)	28 (9.3)	28 (6.5)
Seek information relevant to the care recipient’s needs (medical knowledge)	54 (7.4)	28 (9.3)	26 (6)
Handle financial and legal matters	52 (7.1)	21 (7)	31 (7.2)
Advocate for services	49 (6.7)	22 (7.3)	27 (6.3)
Assist the care recipient to safely transfer in/out of bed, chair wheelchair, toilet and tub/shower	47 (6.4)	21 (7)	26 (6)
Access training resources (nursing training, certified nursing assistant, cardiopulmonary resuscitation, health monitoring, fall prevention, and equipment use)	45 (6.1)	17 (5.6)	28 (6.5)
Manage laundry, prepare meals, perform shopping, and run errands	43 (5.9)	23 (7.6)	20 (4.6)

## Discussion

Our study included a large sample of diverse caregivers who identified common domains and tasks in which they felt inadequately prepared for caregiving tasks and situations. We identified key gaps in caregiver preparedness while exploring similarities and differences between the preparedness concerns of those caring for veterans with versus without dementia. Our findings are quite remarkable. Both groups of caregivers, whether caring for veterans with or without dementia, were very similar in the percentages reporting needs in most domains and tasks. The commonest preparedness gaps were in care coordination, emotional and social support, and advance planning, followed by nursing and health monitoring, personal care, mobility, and household domains.

Caregivers of veterans with dementia in our study were older, provided more hours of care, and were more likely to report high caregiver burden, compared to caregivers of veterans without dementia; this is consistent with current literature [41]. Our results are supported by previous reports showing that veterans with 2 or more self-care needs are similar to veterans with dementia in the high level of care needed and in the type of tasks they need help with [14]. Prior studies have also shown that caregivers of care recipients with dementia report more caregiver strain and less time for social participation [42]. This is supported by our data, in which caregivers of veterans with dementia, versus caregivers of veterans without, were more likely to perceive high caregiver burden, and be interested in strategies to take care of their own well-being [43]. Some potential explanations may be that all veterans are challenging to care for, regardless of their diagnosis. Another explanation is that caregiving, no matter for whom, just has some gaps that caregivers do not feel prepared for. A third possibility might be that the VA does a better job of preparing dementia caregivers, because some caregiver support programs in the VA are more targeted to dementia; for example, Resources for Enhancing All Caregivers’ Health (REACH) VA was initially built for dementia caregivers, even though it has now expanded to other conditions.

Although each caregiver’s path is unique and varies in onset and intensity, the caregiving journey has a somewhat typical trajectory [4,40]. It usually starts with a need for intermittent assistance from caregivers with tasks like transportation, medical appointments, and communicating with medical providers. This evolves over time into a more regular need for assistance with tasks like managing medications and coordinating care, followed by personal care tasks like help with bathing and dressing, and finally, with end-of-life care. Caregiving tasks are not driven by a specific diagnosis but rather by tasks in which caregivers aid with, and are influenced by, the care recipients’ functional capacity, need, and behavioral problems. Therefore, the concept of caregiver preparedness for older adults is complex and evolves with time and changing context. Additionally, since caring for patients with dementia often requires more intensive support over extended periods of time compared to caring for non-dementia patients [22,23], it is perceived to lead to higher levels of burden [26-28] and unmet needs compared to caregivers of other age-related illnesses [27,28]. Yet, our data suggest that the tasks and skills they need over their caregiving journey are somewhat similar, even though the frequency and amount with which a particular skill may be applied may vary.

Caregiver preparedness has been proposed as a prospective target and a potential therapeutic mechanism by which caregiver-focused interventions lead to positive outcomes [3,44]. Prior studies have shown that caregiver preparedness can be modified with interventions that prepare caregivers to take care of their care recipients and themselves. The Department of Veterans Affairs has several programs like the Caregiver Support Program, Building Better Caregivers, and Caregivers FIRST (Finding Important Resources, Support, and Training) [45] that provide support, information, and training to caregivers. These programs also help caregivers navigate the health care system, learn stress and mood management techniques, and teach them skills needed to take care of their veterans. They prepare caregivers and potentially alleviate caregiver stress and burden, which leads to more prosocial outcomes for both caregivers and veterans. Similar programs are also available outside the VA through clinics and other health care systems through agencies like the American Association of Retired Persons, Administration for Community Living, and National Alliance for Caregiving. Yet, data suggest that many caregivers are unaware of these programs [46]. Our results also underscore that while the disease stages, progression, and some expected symptoms may vary by disease and disease types, some crucial elements of caregivers’ preparedness, regardless of the specific condition, include knowledge about caregiver resources, support services, self-care, financial, medical, and legal information.

This study reinforces findings relevant to programs and interventions designed to improve family caregiving competency. First, caregiver intervention programs should focus on direct care and advocacy and coordination. Structured approaches should be used to identify gaps in caregiver preparedness, grouped by domains and tasks, and offer targeted caregiver skills training and information about resources that augment caregiver preparedness, contextualized to their needs, since not all caregiver interventions are the same. Second, programs and interventions to prepare caregivers may gain economies of scale by developing content that covers broad topics relevant to all caregivers, and supplement that by creating diagnosis-specific modules that address disease-specific information and resources. This is a strategy that caregiver interventions have adopted: examples are the RESCUE (Resources & Education for Stroke Caregivers’ Understanding & Empowerment) program [47], which was initially designed to empower caregivers of veterans with stroke, and then expanded to caregivers of veterans postamputation, by adding relevant content, and the REACH VA program which was initially developed for dementia caregivers and is now offered to all caregivers [4,48]. Programs built and accessible on a modular basis per need may increase timely access to more caregivers. Third, while there are available caregiver training programs in the VA and community, there is a lack of awareness of and access to these programs. Large-scale campaigns are needed to amplify awareness regarding these programs and make them more easily available. Health care systems should routinely identify all caregivers at every contact. They should establish standardized processes to identify current and anticipated caregiver preparedness gaps, at a minimum, during changes in care recipients’ care site or health status [5]. They should then provide caregiver outreach with information about available services and assist them in gaining access to caregiver training programs.

Our study has several limitations. First, we defined the dementia status via HCC-51 and HCC-52 codes and treated dementia as a simple yes/no variable, and did not analyze information by dementia subtype, severity, or cognitive/behavioral staging, even though those features heavily shape caregiving tasks and perceived preparedness. Therefore, our results may mask important gradients in needs among caregivers of veterans with mild versus advanced dementia and limit the clinical specificity of the implications for tailoring interventions. However, our objective for this manuscript was to compare preparedness needs of caregivers of dementia versus non-dementia care recipients, rather than by dementia severity. Second, we analyzed short open-ended responses, and not interviews, to classify preparedness gaps into domains. In some instances, it was hard to tease out whether the response expressed a preparedness gap or an unmet need. As an example, the quotes regarding caregiver self-care may be related to the caregivers’ emotional problems or maybe an “objective” call for more assistance and time (unmet need). Third, about two-thirds of the caregivers did not respond to the question we analyzed, and among those who responded, a third simply wrote “no.” Therefore, nonresponse and bias could distort both the reported prevalence and pattern of preparedness gaps we report. We also did not compare the responders to nonresponders, and among the responders, caregivers who reported preparedness needs to those who did not, to test if there is something fundamentally different about those who reported needing help versus those who did not. This would be a future endeavor since it could help us to target those who are likely to need help. Fourth, our sample consists largely of older, mostly female, mostly White, predominantly spousal caregivers of US veterans who are already engaged with the VA system and who responded to a mailed survey. Preparedness needs and needs gaps may look very different for younger caregivers, non-veteran populations, non–English speakers, or families outside integrated health systems. A strength of our study is the large number of caregivers whose responses were tabulated and compared, comprising both caregivers of veterans with and without dementia.

Our study provides evidence for the National Strategy to Support Family Caregivers’ goal to strengthen services and supports [49]. Attention to caregivers' self-perceived preparedness for caregiving may positively influence dyadic outcomes and aging in place. Future research should include a longitudinal examination of outcomes associated with the level and gaps of caregiver preparedness and the need for long-term institutional care.

## Data Availability

The Veterans Affairs (VA) is committed to making data publicly available through its Open Data platform, in accordance with the Open Data Act. Most datasets are accessible; however, personally identifiable information will be protected, and access to sensitive data may require prior approval. For further information regarding data access or specific data requests, please contact the VA Office of Information and Technology.

## Appendix

Multimedia Appendix 1 Domains and tasks for which caregivers wanted to be better prepared, by dementia status.

## Abbreviations

FIRST: Finding Important Resources, Support, and Training
GCF: Geriatrics and Extended Care Data Analysis Center Core Files
HCC: Hierarchical Condition Category
HERO CARE: Home Excellence Resource Outcome Center to Advance, Redefine, and Evaluate Non-Institutional Care
JFI: JEN Frailty Index
REACH: Resources for Enhancing All Caregivers’ Health
RESCUE: Resources & Education for Stroke Caregivers’ Understanding & Empowerment
VA: Veteran Affairs
VISN: Veteran Integrated Service Network
