# Supplementary material for: Self-Perceived Preparedness Needs Among Caregivers of Veterans With and Without Dementia: An Exploratory Study Using Open-Ended Survey Data
Source: JMIR Form Res. 2026 Jan 22;10:e83493. doi: 10.2196/83493 (PMC12877743; doi:10.2196/83493)
Supplement: Multimedia Appendix 1 [file formative_v10i1e83493_app1.docx]

## **Domains and tasks for which caregivers wanted to be better prepared, by dementia status**

| **Domains and Tasks*** | **Overall**  **(N=732)** | | **Caregivers of persons with Dementia**  **(n=301)** | | **Caregivers of persons without dementia**  **(n=431)** | |
| --- | --- | --- | --- | --- | --- | --- |
|  | *n* | *%* | *n* | *%* | *n* | *%* |
| **Care coordination** | **164** | **22.4%** | **67** | **22.3%** | **97** | **22.5%** |
| Navigate and communicate within the health care system (doctors, nurses, social workers, pharmacists, and other health care and long-term services and supports). | 36 | 4.9% | 9 | 3.0% | 27 | 6.3% |
| Seek medical information relevant to the care recipient’s needs | 54 | 7.4% | 28 | 9.3% | 26 | 6.0% |
| Participate in treatment decisions | 3 | 0.4% | 0 | 0.0% | 3 | 0.7% |
| Advocate for services | 49 | 6.7% | 22 | 7.3% | 27 | 6.3% |
| Locate and arrange resources for cash, food, transportation | 32 | 4.4% | 9 | 3.0% | 23 | 5.3% |
| **Personal care** | **65** | **8.9%** | **34** | **11.3%** | **31** | **7.2%** |
| Assist with bathing, dressing, feeding, grooming and personal hygiene | 28 | 3.8% | 14 | 4.7% | 14 | 3.2% |
| Assist with toileting (e.g. getting to and from the toilet, managing incontinence episodes, maintaining continence) | 11 | 1.5% | 9 | 3.0% | 2 | 0.5% |
| Arrange and manage in-home help | 28 | 3.8% | 13 | 4.3% | 15 | 3.5% |
| **Activities of Daily Living (ADLs) – Mobility** | **79** | **10.8%** | **30** | **10.0%** | **49** | **11.4%** |
| Assist the care recipient to safely transfer in/out of bed, chair wheelchair, toilet and tub/shower | 47 | 6.4% | 21 | 7.0% | 26 | 6.0% |
| Home modifications | 16 | 2.2% | 4 | 1.3% | 12 | 2.8% |
| Manage assistive devices such as walkers, canes, or wheelchairs | 14 | 1.9% | 4 | 1.3% | 10 | 2.3% |
| Assist with appropriate mobility and strengthening exercises | 4 | 0.5% | 1 | 0.3% | 3 | 0.7% |
| **Emotional and social support** | **145** | **19.8%** | **56** | **18.6%** | **89** | **20.6%** |
| Provide support in managing stressful situations | 6 | 0.8% | 1 | 0.3% | 5 | 1.2% |
| Manage emotional and behavioral symptoms | 74 | 10.1% | 28 | 9.3% | 46 | 10.7% |
| Recognize and respond to significant changes in the care recipient’s condition | 66 | 9.0% | 28 | 9.3 | 28 | 6.5 |
| **Advance Planning** | **116** | **15.8%** | **49** | **16.3%** | **67** | **15.5%** |
| Participate advance planning (LTC placement "future") | 36 | 4.9% | 19 | 6.3% | 17 | 3.9% |
| End of life services, hospice, palliative | 30 | 4.1% | 11 | 3.7% | 19 | 4.4% |
| Handle financial and legal matters | 52 | 7.1% | 21 | 7.0% | 31 | 7.2% |
| **Nursing and health monitoring tasks** | **94** | **12.8%** | **34** | **11.3%** | **60** | **13.9%** |
| Support treatment adherence | 3 | 0.4% | 1 | 0.3% | 2 | 0.5% |
| Administer medications including oral, topical, and injectable | 6 | 0.8% | 1 | 0.3% | 5 | 1.2% |
| Provide wound care | 10 | 1.4% | 5 | 1.7% | 5 | 1.2% |
| Manage healthy sleep hygiene | 4 | 0.5% | 1 | 0.3% | 3 | 0.7% |
| Manage physical symptoms (e.g. nausea, pain, constipation) | 20 | 2.7% | 5 | 1.7% | 15 | 3.5% |
| Use medical devices to monitor patient’s condition (e.g., blood pressure cuff, pulse oximeter) | 2 | 0.3% | 1 | 0.3% | 1 | 0.2% |
| Manage hearing or vision deficits | 5 | 0.7% | 3 | 1.0% | 2 | 0.5% |
| Access training resources (Nursing training, CNA, CPR, health monitoring, fall prevention, equipment use) | 45 | 6.1% | 17 | 5.6% | 28 | 6.5% |
| **Household tasks** | **58** | **7.9%** | **30** | **10.0%** | **28** | **6.5%** |
| Assist with paying bills and managing finances | 11 | 1.5% | 6 | 2.0% | 5 | 1.2% |
| Manage laundry, prepare meals, perform shopping, and run errands | 43 | 5.9% | 23 | 7.6% | 20 | 4.6% |
| Perform or coordinate home maintenance activities, including odd jobs | 6 | 0.8% | 2 | 0.7% | 4 | 0.9% |
| **Caregiver self-care** | **36** | **4.9%** | **23** | **7.6%** | **13** | **3.0%** |
| Engage in activities that support caregivers own mental, emotional, and physical wellbeing | 28 | 3.8% | 16 | 5.3% | 12 | 2.8% |
| Ask for and accept assistance e.g. respite care | 8 | 1.1% | 7 | 2.3% | 1 | 0.2% |
| **Emergent Situations** | **28** | **3.8%** | **6** | **2.0%** | **22** | **5.1%** |
| Medical Emergencies | 22 | 3.0% | 5 | 1.7% | 17 | 3.9% |
| Weather-related emergencies (Hurricanes, Power outages) | 6 | 0.8% | 1 | 0.3% | 5 | 1.2% |
| *****Tasks do not add up to domains as caregivers could have selected more than one sub-theme.*** | | | | | | |
